# Chest CT imaging features of COVID-19 and its correlation with the PaO_2_/FiO_2_ ratio: a multicenter study in Upper Egypt

**DOI:** 10.1186/s43055-020-00373-1

**Published:** 2020-12-08

**Authors:** Noha M. Attia, Moustafa H. M. Othman

**Affiliations:** Radiology Department, Faculty of Medicine, Assiut University Hospitals, Assiut University, Assiut, 71515 Egypt

**Keywords:** COVID-19, Chest CT, Severity score, PaO_2_/FiO_2_ ratio

## Abstract

**Background:**

The main challenge in managing COVID-19 pandemic is containment of the infection by early detection of the disease and wide dissemination of diagnostic tests with high sensitivity and specificity. Various imaging features were identified by chest CT with different patterns from early disease to diffuse disease with complications. However, CT cannot be performed for all patients. The arterial oxygen partial pressure/fraction of inspired oxygen (PaO_2_/FiO_2_) ratio is evaluated as a rapid and widely available test for the preliminary assessment of disease severity. This study aimed to evaluate the clinical and chest CT imaging features of COVID-19 in Egyptian patients as well as assess the correlation between the chest CT total severity score and the PaO_2_/FiO_2_ ratio to determine its value for preliminary assessment of disease severity.

**Results:**

The most common symptoms were fever (83.2%), dry cough (77%), malaise (68.8%), prolonged headaches (48.5%), and dyspnea (37.6%). CT was positive in 79.2% of the patients. The CT features at presentation were ground-glass opacities only (40%), ground-glass opacities with consolidation (34.4%), and consolidation only (25.6%). Associated findings included crazy paving (17.5%), interlobular septal thickening (47.5%), air bronchogram (15%), bronchiectasis (12.8%), fibrous bands (8.1%), vascular enlargement within the lesion (45.6%), nodules (6.8%), pericardial thickening (5%), and pleural thickening (24.7%). The lesions were typically multilobar (50.5%), posterior (58.1%) with peripheral and central distribution (41.9%). Moderate negative correlation was observed between the CT total lung severity score and PaO_2_/FiO_2_ ratio with *r* = − 0.42 and *P* < 0.001.

**Conclusion:**

The most common pattern of COVID-19 pneumonia in multiple quarantine hospitals was peripheral and central ground-glass opacities with bilateral multilobe posterior involvement and fever was the most common symptom. PaO_2_/FiO_2_ ratio has a moderate negative correlation with the CT total severity score and thus can be used in the preliminary assessment of disease severity.

## Background

Coronavirus disease 2019 (COVID-19) was first reported in Wuhan, China, in December 2019 and it has since spread to many other countries around the world. As a result, on January 30, 2020, the World Health Organization (WHO) declared this outbreak as a global public health emergency. The main challenge was containment of the infection by early detection of the disease and wide dissemination of diagnostic tests with high sensitivity and specificity [1].

The initial evaluations of the real-time reverse transcriptase-polymerase chain reaction (RT-PCR) test performance showed high analytical sensitivity and specificity; however, the test sensitivity was reduced by several factors such as specimen type, specimen collection technique, specimen handling, and stage of infection when the specimen is acquired [2]. False negative RT-PCR tests have also been reported in patients with CT findings of COVID-19 who later on tested positive [3]. Economic factors may also limit the use of the RT-PCR test owing to insufficient specimen collection kits, lab test supplies, and testing equipment. This prevented early widespread testing in many countries which is presumed to be the cause of the rapid transmission of infection by asymptomatic or mildly symptomatic individuals [4].

CT of the chest is sensitive for detection of early parenchymal lung involvement, with precise characterization of the morphology and sites of the lesions. In addition to the early detection of disease progression, CT can confirm the development of complications as acute heart failure from COVID-19 myocardial injury and when performed with intravenous contrast material, pulmonary thromboembolism. It also identifies underlying cardiopulmonary abnormalities that may facilitate risk stratification [1]. However, the overuse of CT on suspected patients can lead to unnecessary radiological exposure and consumption of much needed radiological resources. Several studies have suggested that arterial blood gas values correlate with the extent and morphological characteristics of CT lesions [5, 6]. Therefore, the current study aimed to evaluate the clinical and chest CT imaging features of COVID-19 in Egyptian patients and assess the correlation between the extent of chest CT lesions calculated by the total severity score and the arterial oxygen partial pressure/fraction of inspired oxygen (PaO_2_/FIO_2_) ratio to determine its value for preliminary assessment of disease severity.

## Methods

### Patients

The records of 404 patients who presented to multiple quarantine hospitals from April 15, 2020, to May 15, 2020, with positive real-time reverse transcriptase RT-PCR for COVID-19 were retrospectively collected. There were 218 males (54%) and 186 females (46%), with a mean age 45 years ±18.27 [SD], ranging from 1 to 80 years old.

The clinical symptoms at presentation as well as the arterial blood gas results were recorded and analyzed. The PaO_2_/FiO_2_ ratio was used as a clinical indicator of hypoxemia if < 300 mmHg. Patients also underwent a non-contrast chest CT examination upon admission. All patients were masked and imaged using a dedicated CT machine for suspected and confirmed COVID-19 patients which was cleaned and disinfected after each patient. All patients with history of previous pulmonary disease were excluded from the study. This retrospective study was approved by the institutional review board and patient consent was waived.

### Image acquisition

CT examinations were performed using multi-detector CT scanners (Toshiba Aquilion 16, Toshiba Medical Systems and Siemens SOMATOM Emotion 16, Siemens-Healthineers). The main scanning parameters were as follows: tube voltage = 120 kVp, automatic tube current modulation (30–70 mAs), pitch = 0.99–1.22 mm, matrix = 512 × 512, slice thickness = 5 mm, field of view = 350 mm × 350 mm. All images were then reconstructed with a slice thickness of 0.625–1.250 mm with the same increment. All CT images were acquired at full inspiration with the patient in the supine position and without contrast medium.

### Image analysis

CT images were reviewed by two radiologists in consensus with 15 and 23 years of experience in thoracic imaging. CT images were evaluated as follows: pulmonary lesions (ground-glass opacity (GGO), consolidation or both), shape of the consolidation if present, other pulmonary parenchymal findings (crazy paving, interlobular septal thickening, air bronchogram, bronchiectasis, fibrous bands, or nodules), involved lung lobes (upper, middle, lower), and their number (one/two or more), site of lesions (anterior, posterior, or both), dominant distribution pattern (peripheral, central, or diffuse), and vascular enlargement sign (vascular enlargement inside the lesion resulting from congestion and dilation of small vessels).

The percentage of compromised lung on CT was assessed by the total severity score [7]. Each of the five lung lobes was assessed for degree of involvement and classified as no involvement (0%) = 0, minimal involvement (1–25%) = 1, mild involvement (26–50%) = 2, moderate involvement (51–75%) = 3, or severe involvement (76–100%) = 4. An overall lung total severity score was calculated by summing the scores of the five lobes (range of possible scores, 0–20).

Pleural thickening and effusion were also assessed. Pericardial thickening, defined as pericardial thickness > 2 mm, was confirmed by physical examination, ECG, and/or echocardiography. Mediastinal lymph node enlargement was recorded if the lymph node short-axis diameter was ≥ 10 mm.

### Statistical analysis

The statistical analysis was performed using SPSS version 21.0 (SPSS Inc. Chicago, IL). Continuous variables were displayed as mean ± standard deviation and categorical variables were reported as counts and percentages. Pearson’s correlation was used to analyze the relation between the chest CT total severity score and PaO_2_/FiO_2_ ratio, patients’ age, and sex. *P* value < 0.05 was considered to be statistically significant.

## Results

### Demography and clinical characteristics

The 404 consecutive patients included in the study (218 males and 186 females with a mean age 45 ± 18.27 SD) all had history of contact with COVID-19-positive cases. The most common symptom was fever in 336 patients (83.2%), followed by dry cough in 311 patients (77%) and malaise in 278 patients (68.8%). A total of 196 patients (48.5%) had prolonged headache, 152 patients (37.6%) had dyspnea, 52 patients (12.8%) had sore throat, 22 patients (4%) had pleuritic centrothoracic chest pain, and 11 patients (2.7%) had diarrhea (Table 1). A total of 39 cases (9.6%) were asymptomatic and 35 of them had a normal CT examination while 4 had positive CT findings with severity scores ≤ 3.

**Table 1:** Demography and clinical characteristics of the studied population

**Mean age**	45 ± 18.27 SD
**Range (years)**	1–80
1–10	14 (3.5%)
11–20	28 (6.9%)
21–30	50 (12.4%)
31–40	70 (17.3%)
41–50	62 (15.3%)
51–60	92 (22.8%)
61–70	62 (15.3%)
71–80	26 (6.4%)
**Gender**	
Male	218 (54%)
Female	186 (46%)
**Clinical symptoms**	
Fever	336 (83.2%)
Dry cough	311 (77%)
Malaise	278 (68.8%)
Sore throat	52 (12.8 %)
Headache	196 (48.5%)
Dypsnea	152 (37.6%)
Diarrhea	11 (2.7%)
Pleuritic chest pain	22 (5.4%)

### CT imaging features

**Fig. 1:**
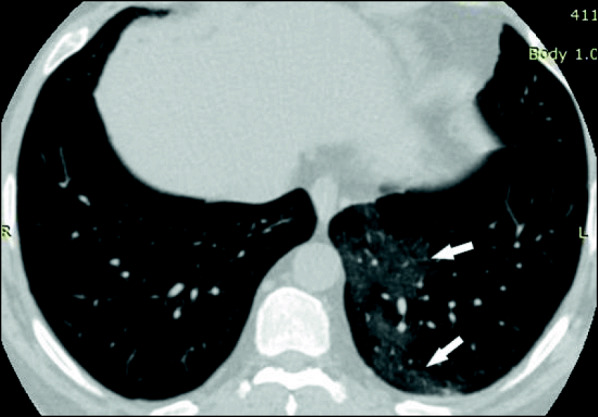
A 65-year-old man who presented with fever, headache, fatigue, and myalgias of 5 days duration. His PaO_2_/FiO_2_ ratio was 410 mmHg. Axial thin-section unenhanced CT image shows peripheral posterior ground-glass opacities in the left lower lobe with a rounded morphology (arrows)

**Fig. 2:**
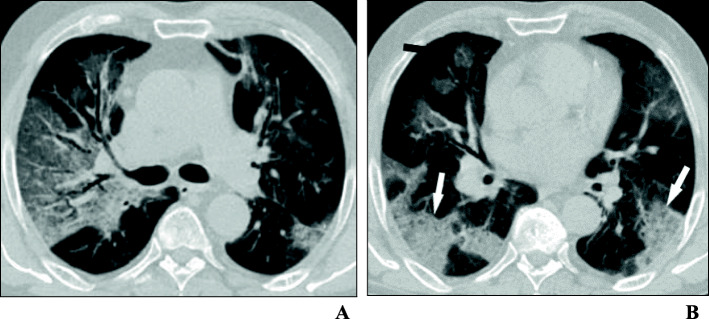
A 66-year-old woman who presented with fever, productive cough, and dyspnea of 10 days duration. Her PaO_2_/FiO_2_ ratio was 250 mmHg. Axial thin-section unenhanced CT images shows **a** a crazy-paving pattern with traction bronchiectasis and **b** ground-glass opacities with round configuration (black arrow) and posterior consolidation (white arrows)

**Fig. 3:**
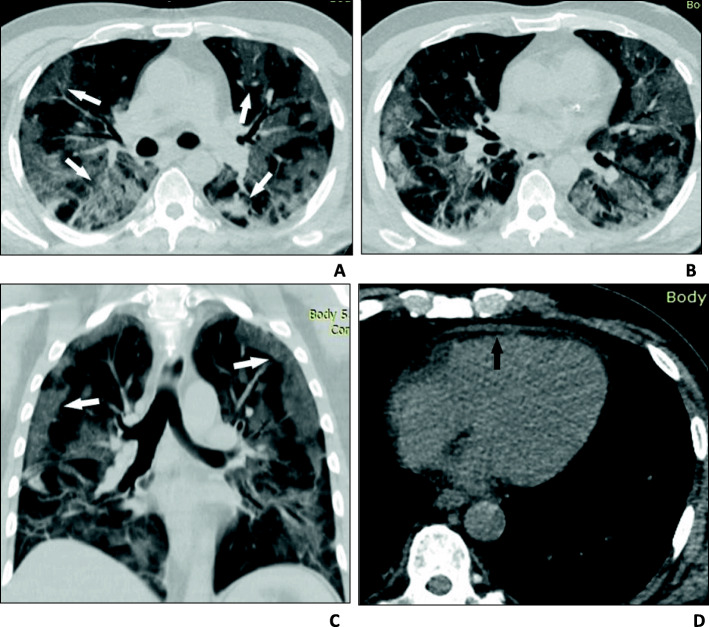
A 60-year-old man who presented with fever and cough followed by centrithoracic chest pain that improved on leaning forward and increased when lying supine and progressive dyspnea requiring admission to the intensive care unit. His PaO_2_/FiO_2_ ratio was 220 mmHg. **a**–**c** Axial and coronal thin-section unenhanced CT scan shows diffuse bilateral confluent and patchy ground-glass and consolidative (white arrows) pulmonary opacities with peripheral and central distribution. **d** Axial image shows thickened pericardium > 2 mm (black arrow) suggesting pericarditis confirmed by echocardiography

**Fig. 4:**
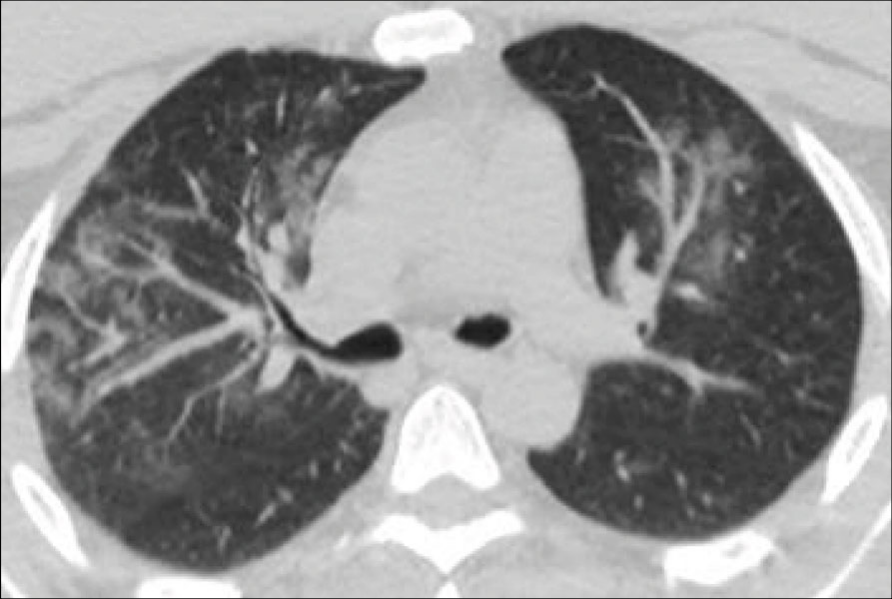
A 40-year-old male presenting with headache, low-grade fever, and cough. His PaO_2_/FiO_2_ ratio was 350 mmHg. Axial thin-section unenhanced CT image shows bilateral upper lobe vascular enlargement within surrounding dense ground-glass opacities with interstitial thickening

Chest CT examination was performed for all patients upon admission. Normal examination was observed in 84 patients (20.8%), while 320 patients (79.2%) had positive findings. Findings at presentation were classified into three main patterns: ground-glass opacities (GGO) only (Fig. 1), consolidation only, and GGO with consolidation (Figs. 2 and 3). The most common lesion at presentation was GGO only which was observed in 128 patients (40%), followed by GGO with consolidation [in 110 patients (34.4%)]. Consolidation only was observed in 82 patients (25.6%). Other associated findings included crazy paving which was observed in 56 patients (17.5%), interlobular septal thickening [152 patients (47.5%)], air bronchogram [48 patients (15%)], bronchiectasis [41 patients (12.8%)], fibrous bands [26 patients (8.1%)], vascular enlargement within the lesion [146 patients (45.6%)] (Fig. 4), and nodules [22 patients (6.8%)] (Table 2).

**Table 2:** Appearance of lesions and associated findings on chest CT

**Appearance of the lesions**		
Ground-glass opacity only	128	40%
Consolidation only	82	25.6%
Ground-glass opacity with consolidation	110	34.4%
Crazy paving	56	17.5%
Air bronchogram	48	15%
Bronchiectasis	41	12.8%
Fibrous bands	26	8.1%
Interlobular septal thickening	152	47.5%
Vascular enlargement within the lesion	146	45.6%
Nodules	22	6.8%
Pleural thickening	79	24.7%
**Mediastinal lymph nodes**	24	7.5%
**Pericardial thickening**	16	5%

**Fig. 5:**
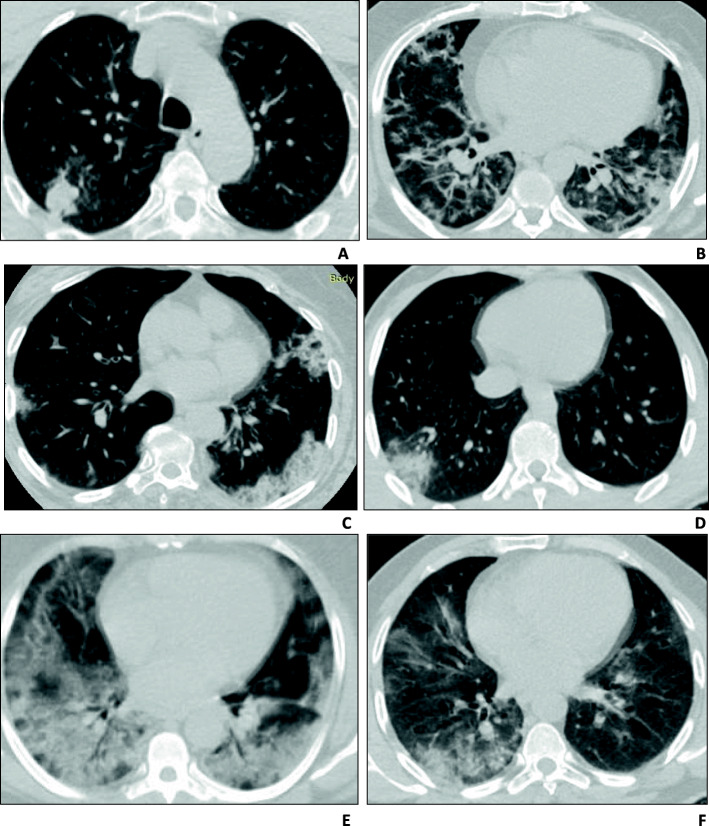
Different shapes of consolidation. **a** Round consolidation. **b** Arcade-like consolidation. **c** Peripheral consolidation parallel to the pleura. **d** Wedge-shaped consolidation. **e** Lobar consolidation with air bronchogram. **f** Irregular patchy consolidation

The shape of consolidation varied among patients. Round consolidation was the most common shape that was observed in 28 (14.5%) of the 192 patients with consolidation, followed by arcade-like consolidation similar to that of organizing pneumonia observed in 25 patients (13%), radial band consolidation in 17 patients (8.9%), wedge-shaped consolidation in 14 patients (7.3%), and lobar consolidation in 13 patients (6.8%) (Fig. 5).

### Lobar involvement and lesion distribution

A total of 280 patients (87.5%) showed involvement of more than one lobe while only 40 patients (12.5%) showed single lobe involvement. The lower lobes were the most commonly affected with lesions seen in 308 patients (96.3%), followed by the upper lobes in 256 patients (80%). The most common pattern of affection was involvement of all lung lobes which was observed in 204 patients (50.5%), followed by involvement of the lower lobes only in 56 patients (13.9%), involvement of the upper and lower lobes in 42 patients (10.4%), involvement of the upper lobes only in 10 patients (2.5%), and involvement of the middle and lower lobes in 8 patients (2%) (Table 3).

**Table 3:** Lobar involvement and lesion distribution on chest CT

**Normal**	84	20.8%
**Number of involved lobes**		
One lobe	40	12.5%
More than one lobe	280	87.5%
**Frequency of lobe involvement**		
Upper lobes	256	80%
Middle lobe	212	66.3%
Lower lobes	308	96.3%
**Pattern of involvement**		
Upper only	10	3.1%
Middle only	0	0%
Lower only	56	17.5%
Upper + middle + lower lobes	204	63.8%
Upper + lower lobes	42	13.1%
Middle + lower lobes	8	2.5%
**Distribution of the lesions**		
Peripheral	134	41.9%
Central	2	0.6%
Peripheral and central	184	57.5%
**Site of the lesions**		
Anterior	10	3.1%
Posterior	186	58.1%
Anterior and posterior	124	38.8%

Regarding the distribution of the lesions, 134 patients (41.9%) had peripheral distribution, 2 patients (0.6%) had central distribution, and 184 patients (57.5%) had both peripheral and central distribution. The lesions were located posteriorly in 186 patients (58.1%), anteriorly and posteriorly in 124 patients (38.7%), and anteriorly in 10 patients (3.1%).

### Quantitative assessment and correlation with PaO_2_/FiO_2_

The percentage of compromised lung was evaluated for each patient by visual assessment using the CT total severity score which ranged from 0 to a maximum of 20, with a mean score of 8.25. Pearson’s correlation was used to analyze the relationship between the CT total severity score and PaO_2_/FiO_2_ ratio, patients’ age, and sex. The recorded PaO_2_/FiO_2_ ratio of all the patients with positive CT findings ranged from 193 to 435. The PaO_2_/FiO_2_ ratio revealed moderate negative correlation with the CT total severity score (*r* = − 0.42 and *P* < 0.001). There was a weak positive correlation between the CT total severity score and age (*r* = 0.33 and *P* < 0.01); however, no significant correlation was found with patients’ sex.

### Associated findings

Mediastinal lymph nodes were found in 24 patients (7.5%). Pericardial thickening defined as pericardial thickness > 2 mm was found in 16 patients (5%) (Fig. 3d). Pleural thickening was found in 79 patients (24.7%). No pericardial or pleural effusion was present in any of the patients (Table 2).

## Discussion

The substantial increase in the number of confirmed and suspected COVID-19 cases has overwhelmed healthcare systems worldwide. Therefore, methods of accurate and rapid diagnosis and triaging are essential to avoid a crisis. Laboratory testing is the standard for diagnosing COVID-19 infection; however, delayed test results and insufficient supply of laboratory kits to cover the increasing number of suspected cases are major drawbacks because early quarantine and treatment are essential for control of the disease [4]. Xie et al. [8] also reported that 5 out of 167 patients (3%) had negative RT-PCR for COVID-19 at initial presentation despite chest CT findings typical of viral pneumonia.

Chest CT appears to be the most sensitive diagnostic tool for COVID-19 pneumonia. Various studies have reported that the morphology and extent of the lung lesions correlate with the severity of the inflammatory process [9, 10]. However, the extent of pulmonary inflammation caused by COVID-19 does not correlate with the severity of clinical manifestations presented at first evaluation in the emergency department [9]. Because CT cannot be performed for all suspected patients, another rapid, inexpensive, and widely available test is needed for the preliminary assessment of patients. Several studies have shown correlation between ABG values and the extent of pulmonary lesions on CT [5, 6]. Furthermore, COVID-19 can be complicated by acute respiratory distress syndrome (ARDS) [11]. Three degrees of ARDS based on the degree of hypoxemia are described: mild (PaO_2_/FiO_2_ ≤ 300 mmHg), moderate (PaO_2_/FiO_2_ ≤ 200 mmHg), and severe (PaO_2_/FiO_2_ ≤ 100 mmHg) [12]. Thus, we used PaO_2_/FiO_2_ values at admission and compared them with the CT total severity score. A moderate negative correlation was observed between the PaO_2_/FiO_2_ ratio and the CT total severity score, *r* = − 0.42, *p* < 0.001, indicating that pulmonary function decreased causing oxygenation impairment as the total severity score increased. This agrees with the results of Turcano et al. [6] who reported that a higher percentage of the lung being involved in the inflammatory process correlated with decreased oxygenation capacity which explained the difficulty of oxygenating the blood, even with high FiO_2_ values. Shang et al. [5] also recorded that the total CT score had a moderate correlation with arterial blood gas indices.

The most common presenting symptoms in this study were fever, cough, and malaise, and other less common symptoms were headache, dyspnea, sore throat, and diarrhea. Shi et al. [13] have also reported fever in 73% of patients and dry cough in 59%. However, 9.7% of the cases in this study were asymptomatic virus carriers while others showed only mild-onset symptoms with no fever and presented only as contacts of positive COVID-19 patients. Twenty-one of the asymptomatic cases (53.8%) showed positive chest CT findings suggesting that chest CT scans or laboratory tests should be done in asymptomatic cases with a history of contact with COVID-19-positive patients to allow early diagnosis and isolation to optimize control of the disease. The number of asymptomatic carriers may be higher than estimated and underdiagnosed due to insufficient laboratory and radiological testing. In the study by Nishiura et al. [14], asymptomatic carriers of COVID-19 have been estimated to comprise 17.9–33.3% of all infected cases. In this study, a normal chest CT scan was found in 84 patients (20.8%) despite positive PCR results. Chung et al. [7] have also reported that 14% of the patients (three out of 21) presented with a normal chest CT scan.

The positive chest CT findings in this study were classified into three main patterns: GGO only, consolidation only, and GGO with consolidation. GGO were the most common lesions at presentation and were observed in 40% of the patients; a slightly lower percentage of patients (34.4%) had associated consolidation. These results were consistent with the study by Li et al. [15] on 51 patients with confirmed COVID-19 infection by nucleic acid testing. Their results showed that only 3.9% of the patients did not have GGO or consolidation which indicated that these lesions are main signs of COVID-19 on CT images. Yoon et al. [16] have also reported that GGO lesions on chest CT without any consolidation were observed in 45% of their cases and in 45–67% of Chinese COVID-19 patients.

The shape of consolidation among our patients varied. Round consolidation was the most common shape observed in 11.9% of the patients, followed by arcade-like consolidation (10.4%), peripheral consolidation parallel to the pleura (7.3%), irregular patchy consolidation with architectural distortion (6.8%), peripheral wedge-shaped consolidation (6.2%), and lobar consolidation (5.4%). Arcade-like consolidation is a typical feature of perilobular fibrosis, which is frequently observed in cryptogenic organizing pneumonia. In 2004, Ujita et al. [17] observed the presence of perilobular fibrosis, with an “arch” pattern, in more than half of the patients with cryptogenic organizing pneumonia, which may be a sequalae of perilobular inflammation.

Associated secondary findings were interlobular septal thickening (152 patients, 47.5%), crazy paving (56 patients, 17.5%), vascular enlargement within the lesion (146 patients, 45.6%), and air bronchogram (48 patients, 15%). No lung cavitation was recorded. Bai et al. [18] described subsegmental vascular enlargement in 59% of the patients with COVID-19 pneumonia versus 22% in those with non-viral pneumonia. Reticular pattern with interlobular septal thickening was reported in several studies as one of the common manifestations of COVID-19 on chest CT, second to GGO and consolidation [13, 19, 20]. With an increase in the disease course, the prevalence of reticular pattern may increase in COVID-19 patients [13]. Posterior location of the opacities was observed in 58.1% of patients while combined peripheral and central distribution was observed in 57.7%. We explain this by late presentation of most of the patients (i.e., more than 7 days after the onset of symptoms). This was also evident in the pattern of affection because involvement of all the lung lobes was the most common pattern seen in 204 patients (50.5%).

Pleural thickening was observed in 79 patients (24.7%). In all cases, it was located adjacent to an area of consolidation or crazy paving indicating pleural involvement in the inflammatory process; however, no pleural effusion was recorded in any of the patients.

On chest CT, the pericardium is best visualized along the right ventricle, due to thin epicardial fat and the vicinity of pulmonary parenchyma along the lateral and posterior left ventricle wall making it difficult to visualize the pericardium on that side. Normal pericardial thickness ranges from 0.7 to 2.0 mm on CT images [21]. In this study, 16 out of 22 patients who presented with pleuritic centrothoracic chest pain (which improved when sitting forward and worsened with supine position in 11 patients) showed pericardial thickness of > 2 mm along the right ventricle suggesting pericarditis. This was confirmed by pericardial rub during physical examination and by the presence of widespread ST elevation on the ECG trace in some patients and T wave flattening in the inferior leads (II, III, and aVF) in others. Pericardial effusion was not observed in any of the patients. The affection of the cardiovascular system in COVID-19 infection was described in a study of 83 patients with severe and critical COVID-19 infection who underwent a CT scan. Chest pain was reported in 6% of the patients and pericardial effusion was observed in 4.8%, suggesting that acute pericarditis could be underdiagnosed [22]. Puntmann et al. [23] conducted a study that included 100 patients who had recovered from COVID-19 disease and underwent cardiac MRI. A total of 78% of the patients had abnormal findings on cardiac MRI, and 22% had pericardial enhancement.

The findings on chest CT may not be specific and can be similar to those in other viral pneumonia. However, together with clinical and laboratory findings, CT of the chest can make an essential contribution to the management of the disease. Among the disadvantages of using CT in the diagnosis of COVID-19 are radiation exposure to the patient, risk of COVID-19 transmission to uninfected healthcare workers and other patients, consumption of personal protective equipment, and need for cleaning and downtime of radiology rooms in resource-constrained environments [4]. In this study, no transmission of COVID-19 to any of the radiology staff was reported as a result of implementing the strict use of personal protective equipment (in those with direct contact with the patients). In addition, downtime caused by cleaning and disinfection was not a problem, because the CT machine was dedicated for COVID-19 patients only, which allowed sufficient time for examining new and follow-up cases.

This study had a few limitations. First, no follow-up imaging was done and thus the dynamic changes of COVID-19 pneumonia which occur over time were not assessed. Furthermore, the outcome of the disease and mortality were not evaluated which would be of great benefit in determining certain prognostic factors as well as their correlation with the PaO_2_/FiO_2_ ratio.

## Conclusion

Currently, chest CT can be used for the early detection of COVID-19 pneumonia, its extent and complications. The typical pattern of COVID-19 pneumonia in Upper Egypt included peripheral and central GGO with bilateral multilobe posterior involvement, and fever was the most common symptom. The moderate negative correlation between the PaO_2_/FiO_2_ ratio and CT total lung severity score suggests that it can be used for the preliminary assessment of disease severity.

## Data Availability

The datasets used and/or analyzed during the current study are available from the corresponding author on reasonable request.

## Abbreviations

CT: Computed tomography
PaO_2_/FIO_2_ ratio: Arterial oxygen partial pressure/fraction of inspired oxygen
GGO: Ground-glass opacity
WHO: World Health Organization
RT-PCR: Real-time reverse transcriptase-polymerase chain reaction
ARDS: Acute respiratory distress syndrome
MRI: Magnetic resonance imaging
